# Chromatin, Non-Coding RNAs, and the Expression of HIV

**DOI:** 10.3390/v5071633

**Published:** 2013-06-28

**Authors:** Jessica N. Groen, Kevin V. Morris

**Affiliations:** 1 School of Biotechnology and Biomolecular Sciences, The University of New South Wales, Sydney, NSW, 2052, Australia; E-Mail: jessica.n.groen@gmail.com; 2 Department of Molecular and Experimental Medicine, The Scripps Research Institute, La Jolla, CA 92037, USA

**Keywords:** HIV, non-coding RNA, chromatin remodeling, latency, reactivation

## Abstract

HIV is a chronic viral infection affecting an estimated 34 million people worldwide. Current therapies employ the use of a cocktail of antiretroviral medications to reduce the spread and effects of HIV, however complete eradication from an individual currently remains unattainable. Viral latency and regulation of gene expression is a key consideration when developing effective treatments. While our understanding of these processes remains incomplete new developments suggest that non-coding RNA (ncRNA) mediated regulation may provide an avenue to controlling both viral expression and latency. Here we discuss the importance of known regulatory mechanisms and suggest directions for further study, in particular the use ncRNAs in controlling HIV expression.

## 1. Introduction

Upon entering a cell, HIV integrates into the genome of the host and essentially functions as an endogenous gene. The ability of HIV to remain dormant, in a relatively quiescent state in the infected cell remains the major barrier in providing an effective cure. Highly active antiretroviral therapy (HAART) is the current standard of care, and although viral load may be reduced to extremely low if not undetectable levels, it is unable to deplete viral reservoirs, and thus the HIV infection remains a lifelong infection. The HIV-1 provirus employs numerous mechanisms to regulate its patterns of gene expression that are vital to the maintenance of latency in cells and therefore its survival. Currently, most of the literature surrounding HIV gene regulation focuses on the role of the long terminal repeat (LTR), and the interactions between of Tat and TAR. This view may be incomplete as new insights suggest a role for long non-coding RNAs (lncRNAs) in the regulation of gene expression patterns in many organisms however their role in HIV is still yet to be elucidated. These ncRNAs have the potential to impact viral lifecycles and pathogenesis in cells and therefore a more holistic understanding of the mechanisms of HIV viral latency and reactivation are paramount if therapeutics are to be developed to eradicate HIV.

## 2. Regulation of HIV-1 Chromatin and Latency

### 2.1. The HIV LTR

**Figure 1:**
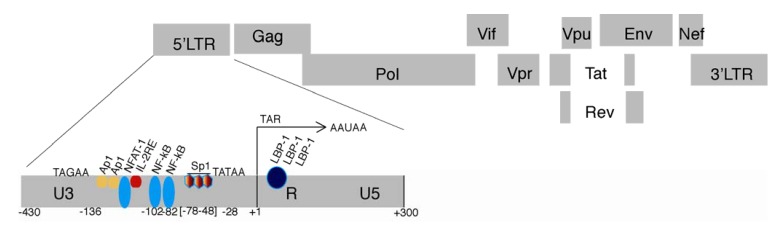
The LTR promoter of HIV. The genomic organization of HIV-1 is shown with lines emphasizing/delineating the 5' LTR/promoter. The transcription factor AP1, NFAT‑1, IL-2RE, NF-kB, Sp1 and LBP-1 binding sites, TATA box, upstream weak transcriptional start TAGAA, Tat activating region (TAR), and the AAUAA poly‑adenylation sites downstream of TAR in the LTR are shown. Note not all transcription factor binding sites are shown.

HIV-1 gene expression is regulated primarily at a transcriptional level by a series of *cis*-acting elements and *trans*-regulatory components located within the LTR at both the 5' and 3' ends of the provirus. The HIV-1 5' LTR comprises of three distinct regions, U3, R and U5, each with different elements involved in transcriptional regulation. The U3 region contains three functional domains, which regulate HIV-1 positive sense transcription: a core promoter region, core enhancer region and a modulatory region (Reviewed in [1]). The promoter region contains numerous transcription factor‑binding sites, most notably, a TATA box and three Sp1 binding sites as well as an initiator element [2,3,4]. Following the promoter region is the core enhancer domain, which contains two NF-κB sites. Interaction between the Sp1 and NF-κB proteins is essential for positive regulation of HIV-1 transcription as the binding of NF-κB alone to the enhancer region is not sufficient for transcriptional activation [5] (Figure 1). 

Many other cellular proteins have also been suggested to have sequence-specific interactions with the modulatory region, contributing to both positive and negative regulation of HIV-1 transcription (Reviewed in [1]). The R region, downstream of the U3 region, contains the transactivation response element (TAR); a viral stem-loop RNA structure which has been well documented to recruit and bind the Tat protein for efficient transcriptional elongation ([6,7], reviewed in [8]). CBP and p300 are coactivating proteins, which acetylate Tat, permitting its association with TAR RNA [9,10,11,12]. The Tat/TAR association synergistically enhances transcriptional efficiency of proviral RNA. The U5 region has also been shown to contain important motifs, which are involved in transactivation of the HIV-1 provirus [13,14]. The capacity of the HIV-1 LTR to mediate complex DNA-protein interactions, which positively and negatively regulate transcription, reveals its importance in the HIV viral lifecycle.

### 2.2. HIV Chromatin Regulation

Post-integration into the host genome, the provirus undergoes packaging into chromatin. Histones are highly conserved proteins, which form the foundation of chromatin structure. Histone proteins associate with DNA to form highly organised and compact structures. This structural unit is termed the nucleosome; and its tight arrangement will act as a barrier for DNA-binding factors such as transcription factors. Limited access to specific proviral sequences impacts the ability of the virus to express genes. Although histones have historically been thought of only as structural elements of the chromatin fibres, it has become evident that they are crucial players in the regulation of gene transcription and have the ability to alter the course of transcription regulation machinery through their histone code [15]. Covalent chemical modifications of the N-terminus of histone tails form the basis of their regulatory capacity and the histone code. These epigenetic modifications such as acetylation, phosphorylation, methylation, ubiquitination and ribosylation are key markers on the protruding histone tails, which may alter chromatin structure and regulation of gene expression (Reviewed in [16]). 

The remodeling and modification of chromatin architecture has since been suggested to be an important factor in the transcriptional regulation of HIV-1 [17,18]. Upon HIV-1 integration into the host genome, the deposition of two nucleosomes, nuc-1 and nuc-0 occurs at two defined regions of the proviral LTR [19]. The deposition of nuc-1 plays an integral role in the suppression of HIV-1 gene expression, ultimately implicating chromatin modification as a key aspect of viral gene repression and latency [20]. 

Histone deacetylases (HDACs) have been associated with HIV-1 transcriptional silencing and viral latency. Cellular transcription factors, such as YY1, LSF and more recently c-Myc, Sp1 and AP-4, have been shown to be involved in the recruitment of HDAC1 to the viral LTR, repressing the HIV-1 promoter and maintaining viral latency [21,22,23]. These changes result in an impaired ability of RNA polymerase II to localise to the HIV-1 promoter and initiate transcription [24]. Following HDAC1 recruitment to the LTR, nucleosome deacetylation and subsequent transcriptional silencing of proviral transcriptional expression have been observed [25]. 

Another epigenetic modification, DNA methylation, has also been identified as a contributing mechanism for viral latency. HIV DNA may become methylated within the viral LTR, which has been associated with transcriptional silencing and the inhibition of the methylation has been shown to result in the reactivation of previously latent HIV [26,27,28]. DNA methylation however, does not seem to be the main silencing mechanism as the effect appears to be reversible and partially methylated promoters may still be reactivated [26].

Likewise, other epigenetic modifications to nucleosomes may play a role in transcriptional activation of HIV-1. It has been shown that the acetylation of histones is correlated with transcriptional activation of the HIV-1 promoter [20]. Acetylation primarily occurs at the lysine residues of the histone tails, which reduces the positive charge of the tails, lowering their affinity for DNA and ultimately causing partial or full disassociation with the DNA [29]. Histone acetyltransferases (HATs) have an opposite action to HDACs and have been known to interact with and be recruited to the viral promoter by the viral protein Tat [9,30,31]. Acetylation of the H3 and H4 histones has been shown to play a strikingly significant role in the onset of viral mRNA transcription in HIV-1 infected cells. In conditions under which the HIV-1 viral promoter is transcriptionally inactive, its activation by Tat correlates with acetylation of H3 and H4 on nuc-0 prior to the onset of viral mRNA production [32]. Furthermore, HAT activity at the promoter sites of genes has been shown to enhance gene transcription although alone it is not able to induce expression of viral expression (Reviewed in [33,34]). 

Essentially, the ability of HIV-1 to alter chromatin structure and epigenetic modifications through cellular and viral transcriptional regulation mechanisms ultimately determines the path of transcriptional activation or viral latency in HIV.

## 3. NcRNAs and Their Role in Genetic Regulation

It has long been accepted that the genome is regulated by a series of aforementioned epigenetic modifications known as the histone code and HIV also appears to be under this mode of gene regulation. The mystery about the concise choreography and execution of the histone code continues to be enigmatic as we delve further into the nuances of the genome. Previous estimates suggested that up to ninety eight per cent of the genome was ‘junk’, however recent observations suggest that this is not the case. It turns out that these so-called ‘junk regions’ of the genome are active and may in fact be assiduous writers of the histone code controlling transcriptional and epigenetic states, among other things. The association of these ncRNAs with chromatin remodeling complexes shapes the future of their bound genes.

### 3.1. LncRNAs, Xist and PTENpg1

In humans, lncRNAs have been found to play a substantial role in chromatin remodeling and gene silencing in both normal cellular function as well as the development of diseases. The most eloquent example of this is the inactivation of the entire X chromosome during cell development. In mammalian females, one copy of the X chromosome is transcriptionally silent for the life of the cell. This is to ensure that the correct ‘dosage’ of expression of the chromosome is achieved with respect to males, who only possess one copy [35]. The silencing of the entire chromosome is mediated by a sophisticated long intergenic noncoding RNA (lincRNA) known as Xist, which is a multiexonic, spliced RNA that possesses its own 5' cap and polyA tail [35,36,37,38]. PRC2 is a large complex with histone methyltransferase activity and is required for the extensive formation of the transcriptionally silent heterochromatin by trimethylation of histone H3 on lysine 27 (Reviewed in [39,40]). The PRC2 complex has been shown to bind RepA; and in turn is bound with Xist to target PRC2 histone methyltransferase activity on the future silent X chromosome [41]. This is the most well recognized example of how a lncRNA can facilitate extensive and essential changes to chromatin and dramatically alter gene expression.

Non-coding RNAs are not only vital for normal development but can also play a role in the development of human diseases such as cancer, Alzheimer’s disease and α-thalassemia [42,43]. The tumor suppressor gene, PTEN, is post-transcriptionally regulated by a lncRNA termed PTEN pseudogene 1 (PTENpg1) [44]. PTENpg1 in turn is modulated by a PTENpg1 encoded asRNA, which exists as one of two isoforms, α and β [45]. The α isoform has been shown to epigenetically silence PTEN transcription while the β isoform interacts with the sense pseudogene to alter the stability and localization of the transcript [45]. The sophistication of directive regulation by lncRNAs allows them to exert substantial control over gene expression and that the equilibrium by which they exist is fundamental for normal cellular functioning.

### 3.2. MicroRNAs (miRNAs) and Small Interfering RNAs (siRNAs); The Role of Dicer and Drosha

LncRNAs are not the only ncRNAs that have a regulatory effect on the genome. MiRNAs and siRNAs are the workhorses of RNA-directed RNA‑processing. The action they exert on processing machinery is termed RNA interference (RNAi). miRNAs are small sequences of about 20–24 nucleotides which regulate gene expression by binding specific cellular mRNA transcripts and directing them to degradation (Reviewed in [46]). The nuclear enzyme, Drosha, and the cytoplasmic enzyme, Dicer are responsible for the maturation of miRNAs in a multistep process (Reviewed in [47]). After the maturation of the miRNA, other components of the RNA induced silencing complex (RISC) are recruited, most of these being nucleases, and degrade target RNA (Reviewed in [48]). The suggested method of targeting particular transcripts comes from the miRNA sequence homology with the mRNA that is to targeted and ultimately degraded [49]. MiRNAs have been found in many species of plants, animals and even in a number of viruses, including the Epstein-Barr Virus (EBV) [50,51,52,53,54,55,56,57,58]. Since viral genomes are extremely compact, utilization of both the positive and negative sense strands seems to be the ideal way to capitalize on their limited regulatory and coding capacity space. 

### 3.3. Mechanisms of RNA-Directed Epigenetic Modification

It is thought that the main mechanism for ncRNA directed epigenetic modifications occurs both in *cis* and in *trans* through specific RNA:DNA and likely RNA:RNA interactions (in the case of *trans*).

*Cis*-acting RNAs are localised to their point of transcription and act directly on surrounding genes on the same chromosome. Acting in *cis*, it is likely that ncRNAs are transcribed with a histone‑modifying protein-binding site, such as the histone methyltransferase PRC2. The PRC2 protein is recruited to the lncRNA while the RNA is still tethered to its transcription site by RNA polymerase II. Transcription factors such as YY1 are then recruited which clamp the hanging protein:ncRNA complex to the chromatin (Reviewed in [59]). The chromatin-modifying protein is then free to induce epigenetic changes such as histone methylation. It has been shown that up to 20% of lincRNAs associate with the histone methyltransferase PRC2 [60].

*Trans*-acting RNAs differ to this as they are not tethered to their site of synthesis but rather are able to move freely and act on genes that are on different chromosomes, some distance away [45]. The ncRNAs are able to recruit histone-modifying proteins and act as a scaffold by which these proteins can associate and form complexes [61]. The mechanism by which *trans*-acting ncRNAs target certain genetic regions to be epigenetically altered is debatable. While there are examples of targeting through sequence homology, whereby the ncRNA forms a DNA:RNA hybrid with the complementary target gene, this may not always be the case. The lncRNA HOTAIR has been shown to interact with up to 850 genome wide target [62] suggesting it is not sequence-specific but that the RNA itself has a structural role in the scaffold. Another potential hypothesis is that the lncRNA interacts with an RNA intermediate at the gene target site. These promoter associated RNAs, named such as they are derived from the promoter of the target gene, have been shown to be mechanistically relevant in antisense RNA (asRNA) directed transcriptional gene silencing [63] and thus have been included as a potential mechanism for lncRNA-directed epigenetic modification. The precise mechanism or combination of mechanisms for *trans*-regulation of epigenetic modification is still yet to be elucidated and may be multifactorial. For a more detailed review on the mechanism of ncRNA-directed epigenetic silencing see Beisel and Paro, 2011 [64].

## 4. HIV and Non-Coding RNAs

It is important to recognize the multiplicity of complex regulatory mechanisms surrounding HIV-1 transcriptional and translational control in order for us to decipher the puzzle of HIV viral latency and reactivation. In addition to chromatin remodeling complexes and the direction of the LTR, other regulatory means are present to control HIV-1 viral expression. 

### 4.1. HIV and asRNAs

Gene expression of retroviruses usually occurs by the transcription of a single transcript, which may be differentiated through splicing events. Transcription is initiated from the 5' end in the LTR, where there are many of the necessary binding sites for transcription factors and regulators of gene expression (Figure 1). With the discovery of new antisense transcripts generated in the HTLV-1 retrovirus [65,66,67], the long-held belief that retroviruses transcribe their genome in a single direction has become redundant and naturally it has been speculated that a similar antisense transcript may exist in other retroviruses such as HIV-1. The possible existence of an antisense protein (ASP) was first proposed by Miller using computational models and analysis of highly conserved regions of HIV-1 proviral DNA [68]. Since then, an asRNA coding for a putative ASP have been characterized [69], however the existence of an actual ASP protein has not yet been determined [70]. Furthermore, it was shown that the regulation this asRNA exerts over the proviral genome may not be due to the ASP, but rather the asRNA itself [71]. Therefore although the existence of the ASP is debatable, there is a growing evidence suggesting a role for the HIV expressed asRNA in controlling HIV-1 expression [72,73,74]. Supporting this notion a recent study by Kobayashi-Ishihara *et al.* has demonstrated that asRNAs may have a regulatory role in HIV-1 viral replication, in particular, regulation of positive sense transcription [75]. These data suggest that the aforementioned asRNA may actually be reclassified as a lncRNA due to its functions in gene regulation, however further studies will be required to elucidate the true function of this HIV expressed asRNA in regulating HIV.

Chromatin remodeling has already been described as an important mechanism for the regulation of viral latency in HIV. Chromatin remodeling in humans is facilitated, at least in part, by lncRNAs that have the ability to form RNA-protein complexes with histone modifying proteins and direct them to the target region (Reviewed in [76,77,78]). Although no lncRNAs have yet been discovered in HIV, their presence in a wide variety of organisms and their role in chromatin remodeling and gene expression would suggest that this is an area of potential interest when considering a possible driving mechanism in HIV latency. 

### 4.2. HIV and miRNAs

In addition to the long asRNAs being utilized by viruses, miRNAs have also been found to be produced by viruses such as EBV and HSV-1. These miRNAs appear to be involved in the post‑transcription regulation of viral mRNA transcripts and also modulating host cell gene expression, essentially with the virus hijacking host miRNA pathways for its own benefit and survival ([55], and reviewed in [79]). MiRNAs are generated from imperfect stem-loop precursors by the enzymes Drosha and Dicer. HIV-1 contains two of these such structures, TAR and RRE [80] so it seems reasonable to extrapolate that it may be able to act in the same way as other viruses, using host machinery to process its own miRNAs. A study by Benasser *et al.* hypothesized, using computational analysis that HIV was also capable of generating up to ten viable viral miRNAs (vmiRNAs) [81]. Further studies have demonstrated that the HIV TAR element is bound by and processed by host-derived Dicer to yield a vmiRNA [82,83,84]. This vmiRNA, processed from the HIV-1 TAR element, was found to inhibit LTR‑driven gene expression, potentially through sequence homology to the TAR element and transcriptional gene silencing [82]. It has also been suggested that HIV may produce vmiRNAs as a part of its pathogenic mechanism. Findings indicate that the HIV-1 proviral genome may have the capacity to produce inhibitory vmiRNAs that interfere with host immunity [85]. These vmiRNAs have been found to theoretically have the ability to block translation of factors such as CD28, CD4 and some interleukins [85]. Supporting this model, HIV-1 has also been shown through deep sequencing analysis, to produce viral small interfering RNAs (vsiRNAs) to further modulate both cellular and viral gene expression [86,87].

## 5. Final Remarks and Future Work

HIV-1 is a complex retrovirus with many regulatory mechanisms in place to control latency, reactivation and gene expression. Although these mechanisms are not yet fully understood, there has been a trend toward widening the scope of the search. Chromatin remodeling complexes and the mechanisms in which they are directed is under particularly intense study due to the increasing amounts of data implicating them as important factors in gene expression. Among the masses of data, lncRNAs seem to be particularly well represented as regulators of chromatin complexes. Further investigation into this area may see the emergence of previously unknown ncRNAs, be they miRNAs, or lncRNAs, in the epigenetic regulation of viral expression and host-virus interactions. 

## References

[1] Pereira L.A., Bentley K., Peeters A., Churchill M.J., Deacon N.J. (2000). A compilation of cellular transcription factor interactions with the HIV-1 LTR promoter. Nucleic Acids Res..

[2] Jones K.A., Peterlin B.M. (1994). Control of RNA initiation and elongation at the HIV-1 promoter. Annu. Rev. Biochem..

[3] Jones K.A., Kadonaga J.T., Luciw P.A., Tjian R. (1986). Activation of the AIDS retrovirus promoter by the cellular transcription factor, Sp1. Science.

[4] Rittner K., Churcher M.J., Gait M.J., Karn J. (1995). The human immunodeficiency virus long terminal repeat includes a specialised initiator element which is required for Tat-responsive transcription. J. Mol. Biol..

[5] Majello B., de Luca P., Hagen G., Suske G., Lania L. (1994). Different members of the Sp1 multigene family exert opposite transcriptional regulation of the long terminal repeat of HIV-1. Nucleic Acids Res..

[6] Graeble M.A., Churcher M.J., Lowe A.D., Gait M.J., Karn J. (1993). Human immunodeficiency virus type 1 transactivator protein, Tat, stimulates transcriptional read-through of distal terminator sequences *in vitro*. Proc. Natl. Acad. Sci. USA.

[7] Wei P., Garber M.E., Fang S.-M., Fischer W.H., Jones K.A. (1998). A Novel CDK9-Associated C-Type Cyclin Interacts Directly with HIV-1 Tat and Mediates Its High-Affinity, Loop-Specific Binding to TAR RNA. Cell.

[8] Brigati C., Giacca M., Noonan D.M., Albini A. (2003). HIV Tat, its TARgets and the control of viral gene expression. FEMS Microbiol. Lett..

[9] Col E., Caron C., Seigneurin-Berny D., Gracia J., Favier A., Khochbin S. (2001). The histone acetyltransferase, hGCN5, interacts with and acetylates the HIV transactivator, Tat. J. Biol. Chem..

[10] Deng L., de la Fuente C., Fu P., Wang L., Donnelly R., Wade J.D., Lambert P., Li H., Lee C.G., Kashanchi F. (2000). Acetylation of HIV-1 Tat by CBP/P300 increases transcription of integrated HIV-1 genome and enhances binding to core histones. Virology.

[11] Kiernan R.E., Vanhulle C., Schiltz L., Adam E., Xiao H., Maudoux F., Calomme C., Burny A., Nakatani Y., Jeang K.T. (1999). HIV-1 tat transcriptional activity is regulated by acetylation. EMBO J..

[12] Ott M., Schnolzer M., Garnica J., Fischle W., Emiliani S., Rackwitz H.R., Verdin E. (1999). Acetylation of the HIV-1 Tat protein by p300 is important for its transcriptional activity. Curr. Biol..

[13] Rabbi M.F., Saifuddin M., Gu D.S., Kagnoff M.F., Roebuck K.A. (1997). U5 region of the human immunodeficiency virus type 1 long terminal repeat contains TRE-like cAMP-responsive elements that bind both AP-1 and CREB/ATF proteins. Virology.

[14] Roebuck K.A., Gu D.S., Kagnoff M.F. (1996). Activating protein-1 cooperates with phorbol ester activation signals to increase HIV-1 expression. AIDS.

[15] Luger K., Richmond T.J. (1998). The histone tails of the nucleosome. Curr. Opin. Genet. Dev..

[16] Strahl B.D., Allis C.D. (2000). The language of covalent histone modifications. Nature.

[17] He G., Ylisastigui L., Margolis D.M. (2002). The regulation of HIV-1 gene expression: The emerging role of chromatin. DNA Cell Biol..

[18] Pumfery A., Deng L., Maddukuri A., de la Fuente C., Li H., Wade J.D., Lambert P., Kumar A., Kashanchi F. (2003). Chromatin remodeling and modification during HIV-1 Tat-activated transcription. Curr. HIV Res..

[19] Verdin E. (1991). DNase I-hypersensitive sites are associated with both long terminal repeats and with the intragenic enhancer of integrated human immunodeficiency virus type 1. J. Virol..

[20] Van Lint C., Emiliani S., Ott M., Verdin E. (1996). Transcriptional activation and chromatin remodeling of the HIV-1 promoter in response to histone acetylation. EMBO J..

[21] Coull J.J., Romerio F., Sun J.M., Volker J.L., Galvin K.M., Davie J.R., Shi Y., Hansen U., Margolis D.M. (2000). The human factors YY1 and LSF repress the human immunodeficiency virus type 1 long terminal repeat via recruitment of histone deacetylase 1. J. Virol..

[22] Jiang G., Espeseth A., Hazuda D.J., Margolis D.M. (2007). c-Myc and Sp1 contribute to proviral latency by recruiting histone deacetylase 1 to the human immunodeficiency virus type 1 promoter. J. Virol..

[23] Imai K., Okamoto T. (2006). Transcriptional repression of human immunodeficiency virus type 1 by AP-4. J. Biol. Chem..

[24] Williams S.A., Chen L.F., Kwon H., Ruiz-Jarabo C.M., Verdin E., Greene W.C. (2006). NF-kappaB p50 promotes HIV latency through HDAC recruitment and repression of transcriptional initiation. EMBO J..

[25] Coull J.J., He G., Melander C., Rucker V.C., Dervan P.B., Margolis D.M. (2002). Targeted derepression of the Human Immunodeficiency Virus type 1 long terminal repeat by pyrrole-imidazole polyamides. J. Virol..

[26] Kauder S.E., Bosque A., Lindqvist A., Planelles V., Verdin E. (2009). Epigenetic regulation of HIV-1 latency by cytosine methylation. PLoS Pathog..

[27] Bednarik D.P., Cook J.A., Pitha P.M. (1990). Inactivation of the HIV LTR by DNA CpG methylation: Evidence for a role in latency. EMBO J..

[28] Bednarik D.P., Duckett C., Kim S.U., Perez V.L., Griffis K., Guenthner P.C., Folks T.M. (1991). DNA CpG methylation inhibits binding of NF-kappa B proteins to the HIV-1 long terminal repeat cognate DNA motifs. New Biol..

[29] Hong L., Schroth G.P., Matthews H.R., Yau P., Bradbury E.M. (1993). Studies of the DNA binding properties of histone H4 amino terminus. Thermal denaturation studies reveal that acetylation markedly reduces the binding constant of the H4 “tail” to DNA. J. Biol. Chem..

[30] Marzio G., Tyagi M., Gutierrez M.I., Giacca M. (1998). HIV-1 tat transactivator recruits p300 and CREB-binding protein histone acetyltransferases to the viral promoter. Proc. Natl. Acad. Sci. USA.

[31] Hottiger M.O., Nabel G.J. (1998). Interaction of human immunodeficiency virus type 1 Tat with the transcriptional coactivators p300 and CREB binding protein. J. Virol..

[32] Lusic M., Marcello A., Cereseto A., Giacca M. (2003). Regulation of HIV-1 gene expression by histone acetylation and factor recruitment at the LTR promoter. EMBO J..

[33] Narlikar G.J., Fan H.Y., Kingston R.E. (2002). Cooperation between complexes that regulate chromatin structure and transcription. Cell.

[34] Cosma M.P. (2002). Ordered recruitment: Gene-specific mechanism of transcription activation. Mol. Cell.

[35] Panning B., Dausman J., Jaenisch R. (1997). X chromosome inactivation is mediated by Xist RNA stabilization. Cell.

[36] Gartler S.M., Goldman M.A. (2001). X-Chromosome inactivation. eLS.

[37] Brown C.J., Ballabio A., Rupert J.L., Lafreniere R.G., Grompe M., Tonlorenzi R., Willard H.F. (1991). A gene from the region of the Human X inactivation centre is expressed exclusively from the inactive X chromosome. Nature.

[38] Penny G.D., Kay G.F., Sheardown S.A., Rastan S., Brockdorff N. (1996). Requirement for Xist in X chromosome inactivation. Nature.

[39] Simon J.A., Kingston R.E. (2009). Mechanisms of Polycomb gene silencing: Knowns and unknowns. Nat. Rev. Mol. Cell Biol..

[40] Plath K., Fang J., Mlynarczyk-Evans S.K., Cao R., Worringer K.A., Wang H., de la Cruz C.C., Otte A.P., Panning B., Zhang Y. (2003). Role of histone H3 lysine 27 methylation in X inactivation. Science.

[41] Zhao J., Sun B.K., Erwin J.A., Song J.J., Lee J.T. (2008). Polycomb proteins targeted by a short repeat RNA to the mouse X chromosome. Science.

[42] Faghihi M.A., Modarresi F., Khalil A.M., Wood D.E., Sahagan B.G., Morgan T.E., Finch C.E., St Laurent G., Kenny P.J., Wahlestedt C. (2008). Expression of a noncoding RNA is elevated in Alzheimer’s disease and drives rapid feed-forward regulation of beta-secretas e. Nat. Med..

[43] Tufarelli C., Stanley J.A., Garrick D., Sharpe J.A., Ayyub H., Wood W.G., Higgs D.R. (2003). Transcription of antisense RNA leading to gene silencing and methylation as a novel cause of human genetic disease. Nat. Genet..

[44] Poliseno L., Salmena L., Zhang J., Carver B., Haveman W.J., Pandolfi P.P. (2010). A coding-independent function of gene and pseudogene mRNAs regulates tumour biology. Nature.

[45] Johnsson P., Ackley A., Vidarsdottir L., Lui W., Corcoran M., Grandér D., Morris K.V. (2013). A pseudogene long non-coding RNA network regulates PTEN transcription and translation in human cells.

[46] Kawasaki H., Wadhwa R., Taira K. (2004). World of small RNAs: From ribozymes to siRNA and miRNA. Differentiation.

[47] Lee Y., Ahn C., Han J., Choi H., Kim J., Yim J., Lee J., Provost P., Radmark O., Kim S. (2003). The nuclear RNase III Drosha initiates microRNA processing. Nature.

[48] Sontheimer E.J. (2005). Assembly and function of RNA silencing complexes. Nat. Rev. Mol. Cell Biol..

[49] Conrad R., Barrier M., Ford L.P. (2006). Role of miRNA and miRNA processing factors in development and disease. Birth Defects Res. C Embryo Today.

[50] Reinhart B.J., Slack F.J., Basson M., Pasquinelli A.E., Bettinger J.C., Rougvie A.E., Horvitz H.R., Ruvkun G. (2000). The 21-nucleotide let-7 RNA regulates developmental timing in Caenorhabditis elegans. Nature.

[51] Lagos-Quintana M., Rauhut R., Lendeckel W., Tuschl T. (2001). Identification of Novel genes coding for small expressed RNAs. Science.

[52] Lau N.C., Lim L.P., Weinstein E.G., Bartel D.P. (2001). An abundant class of tine RNAs with probable regulatory roles in Caenorhabditis elegans. Science.

[53] Mourelatos Z., Dostie J., Paushkin S., Sharma A., Charroux B., Abel L., Rappsilber J., Mann M., Dreyfuss G. (2002). miRNPs: A novel class of ribonucleoproteins containing numerous microRNAs. Genes Dev..

[54] Lagos-Quintana M., Rauhut R., Yalcin A., Meyer J., Lendeckel W., Tuschl T. (2002). Identification of tissue-specific microRNAs from mouse. Curr. Biol..

[55] Pfeffer S., Zavolan M., Grasser F.A., Chien M., Russo J.J., Ju J., John B., Enright A.J., Marks D., Sander C. (2004). Identification of virus-encoded microRNAs. Science.

[56] Umbach J.L., Kramer M.F., Jurak I., Karnowski H.W., Coen D.M., Cullen B.R. (2008). MicroRNAs expressed by herpes simplex virus 1 during latent infection regulate viral mRNAs. Nature.

[57] Sullivan C.S., Sung C.K., Pack C.D., Grundhoff A., Lukacher A.E., Benjamin T.L., Ganem D. (2009). Murine Polyomavirus encodes a microRNA that cleaves early RNA transcripts but is not essential for experimental infection. Virology.

[58] Singh J., Singh C.P., Bhavani A., Nagaraju J. (2010). Discovering microRNAs from Bombyx mori nucleopolyhedrosis virus. Virology.

[59] Lee J.T. (2012). Epigenetic regulation by long noncoding RNAs. Science.

[60] Khalil A.M., Guttman M., Huarte M., Garber M., Raj A., Rivea Morales D., Thomas K., Presser A., Bernstein B.E., van Oudenaarden A. (2009). Many human large intergenic noncoding RNAs associate with chromatin-modifying complexes and affect gene expression. Proc. Natl. Acad. Sci. USA.

[61] Tsai M.C., Manor O., Wan Y., Mosammaparast N., Wang J.K., Lan F., Shi Y., Segal E., Chang H.Y. (2010). Long noncoding RNA as modular scaffold of histone modification complexes. Science.

[62] Gupta R.A., Shah N., Wang K.C., Kim J., Horlings H.M., Wong D.J., Tsai M.C., Hung T., Argani P., Rinn J.L. (2010). Long non-coding RNA HOTAIR reprograms chromatin state to promote cancer metastasis. Nature.

[63] Han J., Kim D., Morris K.V. (2007). Promoter-associated RNA is required for RNA-directed transcriptional gene silencing in human cells. Proc. Natl. Acad. Sci. USA.

[64] Beisel C., Paro R. (2011). Silencing chromatin: Comparing modes and mechanisms. Nat. Rev. Genet..

[65] Mesnard J.M., Barbeau B., Devaux C. (2006). HBZ, a new important player in the mystery of adult T-cell leukemia. Blood.

[66] Gaudray G., Gachon F., Basbous J., Biard-Piechaczyk M., Devaux C., Mesnard J.M. (2002). The complementary strand of the human T-cell leukemia virus type 1 RNA genome encodes a bZIP transcription factor that down-regulates viral transcription. J. Virol..

[67] Larocca D., Chao L.A., Seto M.H., Brunck T.K. (1989). Human T-cell leukemia virus minus strand transcription in infected T-cells. Biochem. Biophys. Res. Commun..

[68] Miller R.H. (1988). Human immunodeficiency virus may encode a novel protein on the genomic DNA plus strand. Science.

[69] Michael N.L., Vahey M.T., d'Arcy L., Ehrenberg P.K., Mosca J.D., Rappaport J., Redfield R.R. (1994). Negative-strand RNA transcripts are produced in human immunodeficiency virus type 1-infected cells and patients by a novel promoter downregulated by Tat. J. Virol..

[70] Torresilla C., Larocque E., Landry S., Halin M., Coulombe Y., Masson J.Y., Mesnard J.M., Barbeau B. (2013). Detection of the HIV-1 minus strand-encoded Antisense Protein and its association with autophagy. J. Virol..

[71] Tagieva N.E., Vaquero C. (1997). Expression of naturally occurring antisense RNA inhibits human immunodeficiency virus type 1 heterologous strain replication. J. Gen. Virol..

[72] Landry S., Halin M., Lefort S., Audet B., Vaquero C., Mesnard J.M., Barbeau B. (2007). Detection, characterization and regulation of antisense transcripts in HIV-1. Retrovirology.

[73] Bentley K., Deacon N., Sonza S., Zeichner S., Churchill M. (2004). Mutational analysis of the HIV-1 LTR as a promoter of negative sense transcription. Arch. Virol..

[74] Peeters A., Lambert P.F., Deacon N.J. (1996). A fourth Sp1 site in the human immunodeficiency virus type 1 long terminal repeat is essential for negative-sense transcription. J. Virol..

[75] Kobayashi-Ishihara M., Yamagishi M., Hara T., Matsuda Y., Takahashi R., Miyake A., Nakano K., Yamochi T., Ishida T., Watanabe T. (2012). HIV-1-encoded antisense RNA suppresses viral replication for a prolonged period. Retrovirology.

[76] Grewal S.I., Moazed D. (2003). Heterochromatin and epigenetic control of gene expression. Science.

[77] Hsieh J., Fire A. (2000). Recognition and silencing of repeated DNA. Annu. Rev. Genet..

[78] Morris K.V. (2011). The emerging role of RNA in the regulation of gene transcription in human cells. Semin. Cell Dev. Biol..

[79] Narayanan A., Kehn-Hall K., Bailey C., Kashanchi F. (2011). Analysis of the roles of HIV-derived microRNAs. Expert Opin. Biol. Ther..

[80] Jeang K.T., Chang Y., Berkhout B., Hammarskjold M.L., Rekosh D. (1991). Regulation of HIV expression: Mechanisms of action of Tat and Rev. AIDS.

[81] Bennasser Y., Le S.Y., Yeung M.L., Jeang K.T. (2004). HIV-1 encoded candidate micro-RNAs and their cellular targets. Retrovirology.

[82] Klase Z., Kale P., Winograd R., Gupta M.V., Heydarian M., Berro R., McCaffrey T., Kashanchi F. (2007). HIV-1 TAR element is processed by Dicer to yield a viral micro-RNA involved in chromatin remodeling of the viral LTR. BMC Mol. Biol..

[83] Bennasser Y., Yeung M.L., Jeang K.T. (2006). HIV-1 TAR RNA subverts RNA interference in transfected cells through sequestration of TAR RNA-binding protein, TRBP. J. Biol. Chem..

[84] Purzycka K.J., Adamiak R.W. (2008). The HIV-2 TAR RNA domain as a potential source of viral-encoded miRNA. A reconnaissance study. Nucleic Acids Symp. Ser. (Oxf.).

[85] Couturier J.P., Root-Bernstein R.S. (2005). HIV may produce inhibitory microRNAs (miRNAs) that block production of CD28, CD4 and some interleukins. J. Theor. Biol..

[86] Schopman N.C., Willemsen M., Liu Y.P., Bradley T., van Kampen A., Baas F., Berkhout B., Haasnoot J. (2012). Deep sequencing of virus-infected cells reveals HIV-encoded small RNAs. Nucleic Acids Res..

[87] Bennasser Y., Le S.Y., Benkirane M., Jeang K.T. (2005). Evidence that HIV-1 encodes an siRNA and a suppressor of RNA silencing. Immunity.

